# A comprehensive evaluation of interaction between genetic variants and use of menopausal hormone therapy on mammographic density

**DOI:** 10.1186/s13058-015-0625-9

**Published:** 2015-08-16

**Authors:** Anja Rudolph, Peter A. Fasching, Sabine Behrens, Ursula Eilber, Manjeet K. Bolla, Qin Wang, Deborah Thompson, Kamila Czene, Judith S. Brand, Jingmei Li, Christopher Scott, V. Shane Pankratz, Kathleen Brandt, Emily Hallberg, Janet E. Olson, Adam Lee, Matthias W. Beckmann, Arif B. Ekici, Lothar Haeberle, Gertraud Maskarinec, Loic Le Marchand, Fredrick Schumacher, Roger L. Milne, Julia A. Knight, Carmel Apicella, Melissa C. Southey, Miroslav K. Kapuscinski, John L. Hopper, Irene L. Andrulis, Graham G. Giles, Christopher A. Haiman, Kay-Tee Khaw, Robert Luben, Per Hall, Paul D. P. Pharoah, Fergus J. Couch, Douglas F. Easton, Isabel dos-Santos-Silva, Celine Vachon, Jenny Chang-Claude

**Affiliations:** Division of Cancer Epidemiology, German Cancer Research Center (DKFZ), Im Neuenheimer Feld 581, D-69120 Heidelberg, Germany; Department of Gynaecology and Obstetrics, University Hospital Erlangen, Friedrich-Alexander University Erlangen-Nuremberg, Comprehensive Cancer Center Erlangen-EMN, Erlangen, Germany; David Geffen School of Medicine, Department of Medicine Division of Hematology and Oncology, University of California at Los Angeles, Los Angeles, CA USA; Centre for Cancer Genetic Epidemiology, Department of Public Health and Primary Care, University of Cambridge, Cambridge, UK; Department of Medical Epidemiology and Biostatistics, Karolinska Institutet, Stockholm, Sweden; Division of Biostatistics, Mayo Clinic, Rochester, MN USA; Department of Radiology, Mayo Clinic, Rochester, MN USA; Department of Health Sciences Research, Mayo Clinic, Rochester, MN USA; Department of Molecular Pharmacology and Experimental Therapeutics, Mayo Clinic, Rochester, MN USA; Institute of Human Genetics, University Hospital Erlangen, Friedrich-Alexander University Erlangen-Nuremberg, Comprehensive Cancer Center Erlangen-EMN, Erlangen, Germany; University of Hawaii Cancer Center, Honolulu, HI USA; Department of Preventive Medicine, Keck School of Medicine, University of Southern California, Los Angeles, CA USA; Cancer Epidemiology Centre, Cancer Council Victoria, Melbourne, Australia; Centre for Epidemiology and Biostatistics, Melbourne School of Population and Global Health, The University of Melbourne, Melbourne, Australia; Prosserman Centre for Health Research, Lunenfeld-Tanenbaum Research Institute of Mount Sinai Hospital, Toronto, Canada; Division of Epidemiology, Dalla Lana School of Public Health, University of Toronto, Toronto, Canada; Department of Pathology, The University of Melbourne, Melbourne, Australia; Lunenfeld-Tanenbaum Research Institute of Mount Sinai Hospital, Toronto, Canada; Department of Molecular Genetics, University of Toronto, Toronto, Canada; MRC Centre for Nutritional Epidemiology in Cancer Prevention and Survival (CNC), University of Cambridge, Cambridge, UK; Clinical Gerontology, Department of Public Health and Primary Care, University of Cambridge, Cambridge, UK; Centre for Cancer Genetic Epidemiology, Department of Oncology, University of Cambridge, Cambridge, UK; Department of Laboratory Medicine and Pathology, Mayo Clinic, Rochester, MN USA; Department of Non-Communicable Disease Epidemiology, London School of Hygiene and Tropical Medicine, London, UK

## Abstract

**Introduction:**

Mammographic density is an established breast cancer risk factor with a strong genetic component and can be increased in women using menopausal hormone therapy (MHT). Here, we aimed to identify genetic variants that may modify the association between MHT use and mammographic density.

**Methods:**

The study comprised 6,298 postmenopausal women from the Mayo Mammography Health Study and nine studies included in the Breast Cancer Association Consortium. We selected for evaluation 1327 single nucleotide polymorphisms (SNPs) showing the lowest *P*-values for interaction (*P*_int_) in a meta-analysis of genome-wide gene-environment interaction studies with MHT use on risk of breast cancer, 2541 SNPs in candidate genes (*AKR1C4*, *CYP1A1-CYP1A2*, *CYP1B1*, *ESR2*, *PPARG*, *PRL*, *SULT1A1-SULT1A2* and *TNF*) and ten SNPs (*AREG*-rs10034692, *PRDM6*-rs186749, *ESR1*-rs12665607, *ZNF365*-rs10995190, 8p11.23-rs7816345, *LSP1*-rs3817198, *IGF1*-rs703556, 12q24-rs1265507, *TMEM184B*-rs7289126, and *SGSM3*-rs17001868) associated with mammographic density in genome-wide studies. We used multiple linear regression models adjusted for potential confounders to evaluate interactions between SNPs and current use of MHT on mammographic density.

**Results:**

No significant interactions were identified after adjustment for multiple testing. The strongest SNP-MHT interaction (unadjusted *P*_int_ <0.0004) was observed with rs9358531 6.5kb 5′ of *PRL.* Furthermore, three SNPs in *PLCG2* that had previously been shown to modify the association of MHT use with breast cancer risk were found to modify also the association of MHT use with mammographic density (unadjusted *P*_int_ <0.002), but solely among cases (unadjusted *P*_int_ SNP×MHT×case-status <0.02).

**Conclusions:**

The study identified potential interactions on mammographic density between current use of MHT and SNPs near *PRL* and in *PLCG2*, which require confirmation. Given the moderate size of the interactions observed, larger studies are needed to identify genetic modifiers of the association of MHT use with mammographic density.

**Electronic supplementary material:**

The online version of this article (doi:10.1186/s13058-015-0625-9) contains supplementary material, which is available to authorized users.

## Introduction

High mammographic density for a woman’s age and body mass index (BMI) - meaning large radio-dense fibro-glandular areas that appear white or bright on a mammogram - is considered an established risk factor for breast cancer. The association with mammographic density seems to be present for risk of both estrogen receptor negative and estrogen receptor positive breast cancer [1, 2]. Mammographic density changes over lifetime and generally declines with age [3]. The decline of mammographic density with age may seem paradoxical for a risk factor, as breast cancer risk generally increases with age. This contradiction may be resolved when regarding mammographic density as a risk factor cumulating over time [4, 5]. Heritable factors have been estimated to explain up to 63 % of variation in mammographic density, implicating a strong genetic component [6, 7]. Also, recent evidence suggests that the biological attributes leading to greater mammographic density and development of breast cancer have common predisposing genes [8–11].

The use of tamoxifen is associated with a decrease in mammographic density [12–14], whereas the use of menopausal hormone therapy (MHT) is associated with higher mammographic density [15–17]. To date, the biological mechanisms by which MHT use influences mammographic density are largely unknown [18]. On the other hand, it is established that the use of MHT is associated with increased breast cancer risk and that the health risks of extended MHT use may exceed the benefits [19]. However, approximately 5 % of women aged 40 years or older reported current use of oral MHT in the US National Health and Nutrition Examination Survey in 2009–2010 [20]. In Europe, similar proportions of women aged 45–69 years were reported to currently use MHT in 2010 according to estimations based on sales data [21].

To better understand the relationship between MHT use and mammographic density, several studies investigated whether polymorphisms in candidate genes related to hormone metabolism, nuclear hormone receptors and growth factors are associated with mammographic density and whether these polymorphisms show a statistical interaction with MHT use, i.e., modify the association between MHT and mammographic density [22–28]. Most studies did not identify any significant interaction, but the null findings can also be attributed to the generally small sample sizes. Two studies observed potential gene-environment interactions between MHT use and single nucleotide polymorphisms (SNPs) in *AKR1C4*, *CYP1A1-CYP1A2*, *CYP1B1*, *ESR2*, *PPARG*, *PRL*, *SULT1A1-SULT1A2* and *TNF*, which warrant confirmation by further studies [24, 28]. We therefore conducted a comprehensive replication analysis in the largest study available to date using genotypes and imputed genotypes of SNPs located in or near these genes.

Candidate gene association studies might have missed gene-environment interactions with genes not selected for study. We previously conducted a meta-analysis of four case-only genome-wide gene-environment interaction studies to identify genetic variants that modify the association of MHT use with breast cancer risk [29]. We hypothesized that these common variants may also modify the association between MHT use and mammographic density. Therefore, we selected the most significant SNPs from the genome-wide G×MHT interaction studies of breast cancer risk for assessment of interaction with MHT use on mammographic density. Furthermore, we included ten variants associated with age-adjusted and BMI-adjusted percent density, dense area or non-dense area at the genome-wide significance level (*ZNF365-*rs10995190 [30], 12q24-rs1265507 [31], and *AREG*-rs10034692, *PRDM6*-rs186749, *ESR1*-rs12665607, 8p11.23-rs7816345, *LSP1*-rs3817198, *IGF1*-rs703556, *TMEM184B*-rs7289126, and *SGSM3*-rs17001868 [10]).

## Materials and methods

### Study sample

The analysis was carried out on pooled data from a nested case–control study of the Mayo mammography health study (MMHS), and from five case–control studies (Australian breast cancer family study (ABCFS), Bavarian breast cancer cases and controls (BBCC), Mayo clinic breast cancer study (MCBCS), Ontario familial breast cancer registry (OFBCR), and the Singapore and Sweden breast cancer study (SASBAC)), two nested case–control studies (Melbourne collaborative cohort study (MCCS) and the Multi-ethnic cohort (MEC)), one cohort study (European prospective investigation into cancer and nutrition (EPIC)) and one family study (Sisters in breast screening study (SIBS)), participating in the Breast Cancer Association Consortium (BCAC) [32]; EPIC and SIBS samples are part of SEARCH [33] within BCAC). Details on study design and recruitment are provided in Additional file 1: Table S1. All participants signed informed consent and the studies were approved by the relevant ethics committees. The names of the individual approving ethics committees for each study can be found within the Acknowledgements section.

Participants were eligible for this study if information on mammographic density, relevant covariates and SNP genotypes was available and if they were of European descent and postmenopausal at the time of mammography. Ancestry informative principal components were used to define probable ethnic ancestry, with the exception of MMHS, where ethnicity was self-reported or abstracted from clinical records. In total, 6,298 individuals were included in the analysis.

### Density measures and exposure definitions

Each study collected radiographs of mammograms from participants, either in the mediolateral oblique or craniocaudal view. The radiographs were digitized and percent density, dense area, and non-dense area measures were obtained using one of two similar semi-automated methods, Cumulus [34] and Madena [35]. Measurements using the two methods have been found to be highly correlated (Pearson correlation coefficient of 0.86) [36].

Information on relevant exposures such as age, MHT use and BMI was collected individually by each study. The date of the mammogram was the reference date used for all exposure definitions. Ever use of MHT was defined as use of any type of MHT. Women were categorized as current users when using MHT at the date of mammography, former users if they had used MHT previously and never users if they had never used MHT. As part of the DENSNP project [9], individual study data were centrally quality checked and harmonized at the Mayo Clinic, Rochester, Minnesota.

### SNP selection, genotyping and imputation

Using a meta-analysis of four case-only genome-wide gene-environment interaction studies on the association between MHT use, and overall and lobular breast cancer risk [29], 5,000 SNPs were initially selected based on evidence of interaction with MHT. For each SNP, the lower *P* value for interaction (*P*_int_) from the results for overall and lobular breast cancer was used. After exclusion of SNPs with minor allele frequency (MAF) <0.05, *P* <0.05 for Cochran’s *Q* or *I*^2^ ≥30% for study heterogeneity and the availability of the respective SNP data in fewer than two case-only studies, 4,421 SNPs remained. Of these, the 1,391 SNPs showing *P*_int_ <0.003 were selected for inclusion in a custom Illumina iSelect genotyping array (iCOGS). The iCOGS data were centrally quality controlled after genotyping, which led to the exclusion of 56 SNPs. SNP exclusion criteria were a call rate of <95 %, monomorphism, deviation from Hardy-Weinberg equilibrium with *P* <1.0 × 10^−7^ and concordance in duplicate samples <98% [37]. We additionally excluded seven SNPs with MAF <0.05 in our dataset.

We additionally identified 5,457 SNPs located in or 50 kb around *AKR1C4*, *CYP1A1-CYP1A2*, *CYP1B1*, *ESR2*, *PPARG*, *PRL*, *SULT1A1-SULT1A2* and *TNF* for replication analysis, which were available through iCOGS genotyping and imputation. Of these, 2,541 SNPs were available in BCAC studies and MMHS. For MMHS, the 1000 Genomes Phase I version 3 March 2012 release of the reference panel was used for imputation. Imputation was done using SHAPEIT [38] and IMPUTE.V2 [39]. Imputed SNPs were excluded if the imputation accuracy *r*^2^ was <0.3. For BCAC studies, imputation was conducted centrally in Cambridge, using the same methods and reference panel that were used for MMHS. Imputed SNPs with MAF <0.05 or imputation accuracy *r*^2^ <0.3 were excluded from analysis.

In total, we analyzed 1,327 genotyped SNPs selected based on the genome-wide interaction studies, plus 121 genotyped SNPs and 2,420 imputed SNPs located in or near *AKR1C4*, *CYP1A1-CYP1A2*, *CYP1B1*, *ESR2*, *PPARG*, *PRL*, *SULT1A1-SULT1A2* and *TNF*, for replication analysis. In addition, genotypes of *AREG*-rs10034692, *PRDM6*-rs186749 (imputed), *ESR1*-rs12665607, *ZNF365*-rs10995190, 8p11.23-rs7816345, *LSP1*-rs3817198, *IGF1*-rs703556, 12q24-rs1265507, *TMEM184B*-rs7289126, and *SGSM3*-rs17001868 were analyzed.

### Statistical analysis

The mammographic density variables were square-root-transformed to meet the model assumption of normality of the error distribution. Associations between SNPs and mammographic density measures (first, percent density and second, dense area and non-dense area) were assessed using multiple linear regression models (PROC MIXED, SAS 9.2). All models were adjusted for study, age (continuous), case status (breast cancer case/non-case), BMI (continuous), former MHT use (yes/no), number of pregnancies (continuous) and 15 ancestry informative principal components (continuous) that had been constructed previously [40]. For MMHS, the principal components were not available and have been set to 0. SNP genotypes were coded according to an additive model (0, 1, 2 alleles) and entered as a continuous variable. For imputed SNPs, the estimated allele dosage was entered (values between 0 and 2). To evaluate interactions between SNPs and current MHT use on mammographic density measures, we included a respective interaction term in the models. All statistical tests were two-sided. To account for the number of tests performed, we calculated adjusted *P* values according to a false discovery rate (FDR) of 10 %, applying the method described by Benjamini and Hochberg [41]. We report estimates from analyses pooling individual study data, and assessed between-study heterogeneity by calculating Cochrane’s *Q* and *I*^2^ based on the per-study estimates, using the package *meta*, version 2.1-2 within the R software, version 2.15.2.

For illustration, the association between current use of MHT and mammographic density measures stratified by SNP genotypes was calculated. For imputed SNPs, allele dosages <0.5 of the coding allele were translated into an imputed homozygous reference genotype. Likewise, allele dosages ≥0.5 and <1.5 were translated into an imputed heterozygous genotype and allele dosages ≥1.5 into an imputed homozygous non-reference genotype.

Because SNPs selected for this study were previously found to have a potential multiplicative statistical interaction with use of MHT on breast cancer risk, we evaluated whether interactions on mammographic density differed for cases and non-cases by entering a three-way interaction term (SNP × MHT × case status) into multiple regression models.

Potential functional implications of selected SNPs were assessed using HaploReg v2 [42] and the University of California Santa Cruz (UCSC) genome browser [43]. Linkage disequilibrium (LD) between SNPs of interest was assessed using LD information from the 1000 Genomes Project within HaploReg v2.

## Results

The characteristics of the study population according to mammographic density measurements are described in Table 1. The adjusted mean percent mammographic density was higher in younger women, in women with a lower BMI, and in nulliparous women compared to women with one or more pregnancies. Women currently using MHT compared to never users or former users had a higher adjusted mean percent density as did women diagnosed with breast cancer compared to non-cases. Additional file 2: Table S2 shows the characteristics by study.

**Table 1:** Mammographic density measurements by known breast cancer risk factors, mammographic projection, and case status at time of mammography

Characteristic	Number (%)	PD, mean (95 % CI)^a,b^	Dense area, cm^2^, mean (95 % CI)^a,b^	Non-dense area, cm^2^, mean (95 % CI)^a,b^
Total	6,298 (100.0)	18.1	28.5	143.1
Age, years				
<50	190 (3.0)	25.9 (23.8, 28.0)	32.9 (29.8, 36.1)	98.4 (92.4, 104.5)
≥50 to <60	1,948 (30.9)	21.3 (20.4, 22.2)	30.8 (29.4, 32.3)	114.9 (111.8, 118.0)
≥60 to <70	2,939 (46.7)	17.6 (16.8, 18.4)	27.0 (25.7, 28.3)	127.9 (124.7, 131.0)
≥70 to <80	1,101 (17.5)	16.7 (15.7, 17.7)	25.8 (24.2, 27.5)	131.9 (128.0, 136.0)
≥80	120 (1.9)	14.3 (12.3, 16.4)	22.1 (18.9, 25.5)	135.8 (126.9, 145.0)
BMI, kg/m^2^				
<25	2,488 (39.5)	25.0 (24.0, 25.9)	30.0 (28.7, 31.4)	85.9 (83.3, 88.5)
≥25 to <30	2,244 (35.6)	17.6 (16.8, 18.4)	27.9 (26.6, 29.3)	129.0 (125.7, 132.4)
≥30 to <35	1,088 (17.3)	13.3 (12.4, 14.1)	25.5 (24.0, 27.1)	170.0 (165.5, 174.6)
≥35	478 (7.6)	10.2 (9.3, 11.2)	23.0 (21.1, 24.9)	211.3 (204.7, 217.9)
MHT use				
never	2,885 (45.8)	16.2 (15.6, 16.9)	23.7 (22.7, 24.8)	128.3 (125.6, 131.1)
former	2,100 (33.3)	17.5 (16.8, 18.3)	26.0 (24.8, 27.3)	125.7 (122.8, 128.7)
current	1,313 (20.9)	19.9 (19.0, 20.8)	29.7 (28.2, 31.2)	121.5 (118.2, 124.9)
Parity				
nulliparous	775 (12.3)	22.3 (21.1, 23.5)	32.7 (30.9, 34.6)	117.5 (113.6, 121.4)
1 full-term pregnancy	848 (13.5)	20.2 (19.1, 21.3)	31.0 (29.2, 32.9)	124.0 (120.0, 128.0)
2 full-term pregnancies	2,369 (37.6)	18.3 (17.5, 19.1)	27.5 (26.2, 28.9)	126.2 (123.1, 129.4)
≥3 full-term pregnancies	2,306 (36.6)	17.2 (16.5, 18.0)	25.3 (24.0, 26.6)	123.2 (120.2, 126.3)
Mammographic view^c^				
MLO	3,454 (54.8)	14.6 (14.1, 15.1)	23.3 (22.4, 24.2)	139.3 (137.1, 141.6)
CC	2,844 (45.2)	19.0 (18.5, 19.5)	29.0 (28.1, 30.0)	122.6 (120.7, 124.6)
Case status				
non-case	4,054 (64.4)	17.0 (16.2, 17.8)	25.7 (24.5, 27.0)	128.4 (125.3, 131.6)
case	2,244 (35.6)	20.4 (19.6, 21.3)	30.0 (28.7, 31.4)	118.9 (116.0, 121.8)

^a^Back-transformed. ^b^Adjusted for study, reference age, use of menopausal hormone therapy (*MHT*), body mass index (*BMI*) and number of pregnancies. ^c^In each study mammographs were taken in either the mediolateral oblique (*MLO*) or craniocaudal (*CC*) projection. *PD* percent density

**Fig. 1:**
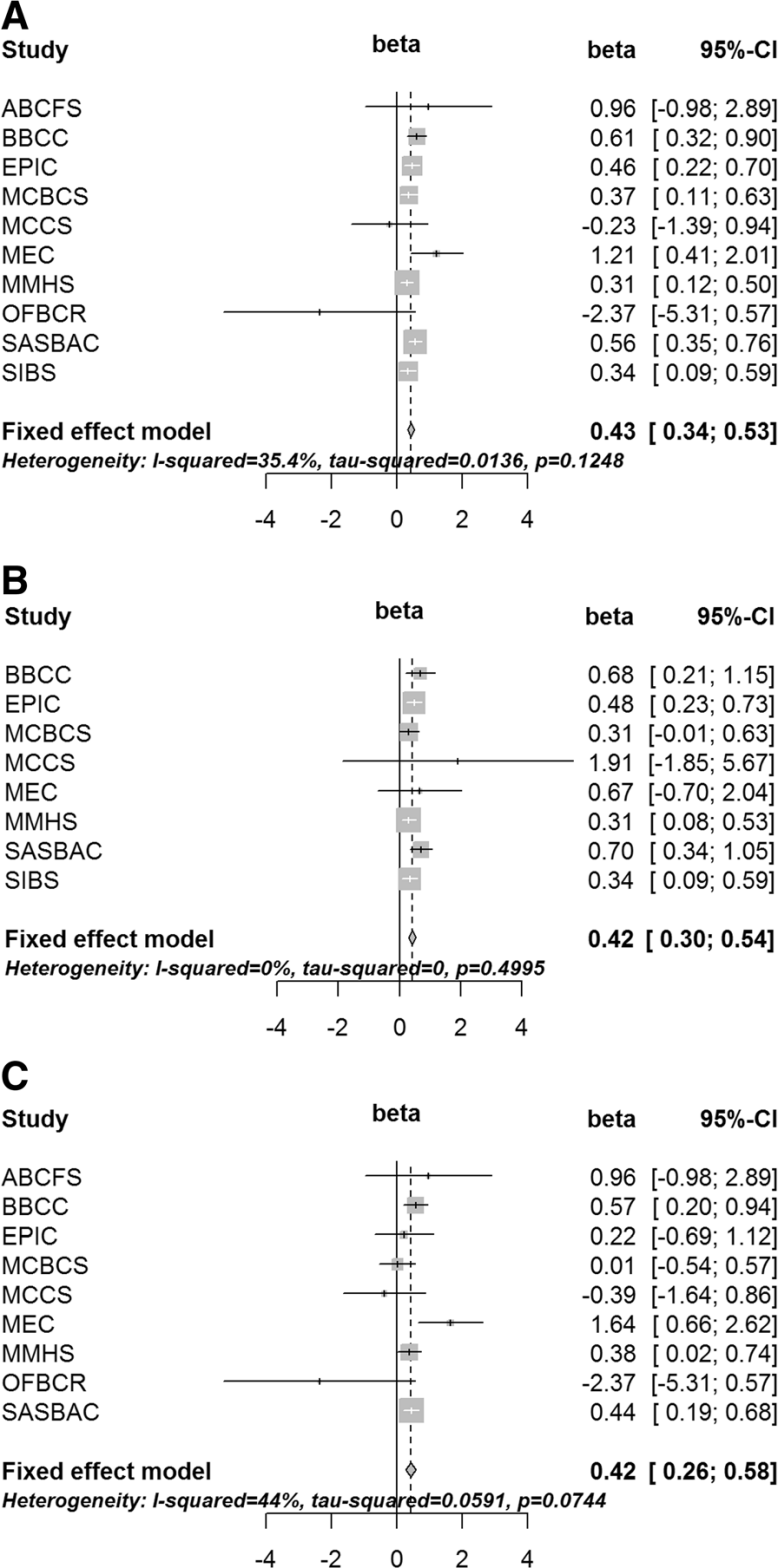
Meta-analyses of study estimates for association of current use of menopausal hormone therapy with square-root-transformed percent mammographic density analyzing all study subjects (**a**), non-cases (**b**) and cases (**c**). *ABCFS* Australian breast cancer family study, *BBCC* Bavarian breast cancer cases and controls, *EPIC* European prospective investigation into cancer and nutrition, *MCBS* Mayo clinic breast cancer study, *MCCS* Melbourne collaborative cohort study, *MEC* Multi-ethnic cohort, *MMHS* Mayo mammography health study, *OFBCR* Ontario familial breast cancer registry, *SASBAC* Singapore and Sweden breast cancer study, *SIBS* Sisters in breast screening study

The beta value from fixed effect meta-analysis of the association between current use of MHT and square-root-transformed percent density was 0.43 (95 % CI 0.34, 0.53). There was some indication of between-study heterogeneity (*P* value for heterogeneity = 0.12, *I*^2^= 35.4 %), which was attributable to one study (OFBCR). The beta value for current use of MHT was very similar when analyzing solely cases (beta = 0.42, 95 % CI 0.26, 0.58) or non-cases (beta = 0.42, 95 % CI 0.30, 0.54). The forest plots for the whole study sample, non-cases and cases, respectively are displayed in Fig. 1.

We did not identify significant associations between the analyzed SNPs and percent density (all FDR adjusted *P* values >0.1). The top ten SNPs associated with percent density are shown in Table 2. The association with percent density with the lowest *P* value was observed for rs181042206 on chromosome 15 (5.2kb 3′ of *CYP1A1*, beta = −0.10, 95 % CI −0.16, −0.04, *P* = 0.0006). The association was not heterogeneous between studies (*P* value for heterogeneity = 0.32, *I*^2^ = 13.6 %). For the components of percent density, rs181042206 was more strongly associated with non-dense area (beta = 0.14, 95 % CI 0.05, 0.23, *P* = 0.002) than dense area (beta = −0.10, 95 % CI −0.18, −0.02, *P* = 0.01).

**Table 2:** Ten SNPs with the lowest *P* values for association with percent mammographic density

SNP	SNP type	Chr	Gene, RefSeq	Feature	Percent density^a^			Dense area, cm^2a^		Non-dense area, cm^2a^	
					Beta^b^ (95 % CI)	*P*	*P*_*adj*_^c^	Beta^b^ (95 % CI)	*P*	Beta^b^ (95 % CI)	*P*
rs181042206	Imputed	15	5.2kb 3′ of CYP1A1		−0.10 (−0.16, −0.04)	0.0006	2.51	−0.10 (−0.18, −0.02)	0.01	0.14 (0.05, 0.23)	0.002
rs12258125	Genotyped	10	272kb 5′ of ANKRD30A		0.09 (0.04, 0.14)	0.0008	1.53	0.10 (0.03, 0.17)	0.004	−0.07 (−0.14, 0.00)	0.07
rs11616761	Genotyped	13	CDC16	Intronic	0.14 (0.05, 0.23)	0.0022	2.83	0.18 (0.06, 0.29)	0.004	−0.11 (−0.24, 0.02)	0.09
rs4632572	Genotyped	3	254kb 5′ of ALCAM		0.08 (0.03, 0.14)	0.0027	2.59	0.14 (0.07, 0.21)	0.0002	0.00 (−0.08, 0.08)	0.96
rs11896627	Genotyped	2	241kb 5′ of NCKAP5		−0.08 (−0.13, −0.03)	0.0032	2.49	−0.10 (−0.17, −0.03)	0.003	0.03 (−0.05, 0.10)	0.51
rs2446585	Genotyped	10	FRMD4A	Intronic	−0.07 (−0.12, −0.02)	0.0038	2.46	−0.08 (−0.15, −0.02)	0.01	0.07 (−0.01, 0.14)	0.07
rs273352	Genotyped	18	MAPRE2	Intronic	−0.08 (−0.13, −0.02)	0.0058	3.22	−0.08 (−0.15, −0.01)	0.04	0.10 (0.02, 0.18)	0.01
rs477705	Genotyped	18	MAPRE2	Intronic	−0.08 (−0.13, −0.02)	0.0058	2.82	−0.08 (−0.15, −0.01)	0.03	0.10 (0.02, 0.18)	0.01
rs10776775	Genotyped	1	LOC100287722	Intronic	−0.07 (−0.12, −0.02)	0.0062	2.64	−0.09 (−0.15, −0.02)	0.009	0.01 (−0.06, 0.08)	0.78
rs2057469	Genotyped	9	RAB14	3′-UTR	−0.07 (−0.12, −0.02)	0.0066	2.55	−0.08 (−0.15, −0.01)	0.02	0.08 (0.00, 0.16)	0.04

^a^Square-root-transformed. ^b^Adjusted for study, reference age, case status, current use of menopausal hormone therapy (MHT), former use of MHT, body mass index, number of pregnancies and principal components. ^c^Adjusted *P* value, calculated by multiplying *P* value by number of tests (here n = 3,868) and dividing by assigned rank. *SNP* single nucleotide polymorphism, *Chr* chromosome, *UTR* untranslated region

There was no significant interaction between current use of MHT and the SNPs after correcting for multiple testing (all adjusted *P*_int_ >0.1). The ten SNPs showing the lowest *P*_int_ on percent mammographic density are displayed in detail in Additional file 3: Table S3. Results for all investigated SNPs can be found in Additional file 4: Tables S6a to S6c. Table 3 shows associations between current MHT use and mammographic density stratified by these SNPs. The imputed variant rs9358531 on chromosome 6 (6.5kb 5′ of *PRL*) had the strongest interaction (*P*_int_ = 0.0004). Current use of MHT was associated with percent mammographic density in women with imputed G/G genotype of rs9358531 with beta = 0.69, 95 % CI 0.49, 0.89, *P* = 1.4 × 10^−11^. The association was less strong in women with imputed T/T genotype (beta = 0.23, 95 % CI 0.08, 0.38, *P* = 2.5 × 10^−03^). We did not observe study heterogeneity for this interaction (*P* value for heterogeneity = 0.75, *I*^2^ = 0 %). The interaction between rs9358531 and current use of MHT on percent density was also similar in cases and non-cases (in cases: beta_int_ = 0.28, 95 % CI 0.06, 0.51, *P*_int_ = 0.01; in non-cases: beta_int_ = 0.21, 95 % CI 0.05, 0.37, *P*_int_ = 0.01; *P*_int_ SNP × MHT × case status = 0.60). Six SNPs among the ten SNPs showing the lowest *P*_int_ are in LD with rs9358531 (rs9356811, rs10946546, rs9393273, rs12525289, rs12199382, rs12524161) with *r*^2^ ranging from 0.50 to 0.80. A genotyped SNP (rs1935007) in moderate LD with rs9356811 (*r*^2^ = 0.42) had a similar but weaker interaction (beta_int_ = 0.20, 95 % CI 0.07, 0.32, *P*_int_ = 0.002) compared to the imputed SNPs in the region. Furthermore, we observed a potential interaction between MHT and a genotyped SNP on chromosome 13 (rs9542456, 505 kb 3′ of *ATXN8OS*). In women carrying the G/G genotype, current use of MHT was associated with percent mammographic density (beta = 0.59, 95 % CI 0.46, 0.73, *P* = 4.7 × 10^−18^). This association was attenuated in women carrying the A/A genotype (beta = 0.22, 95 % CI −0.01, 0.45, *P* = 0.06, *P*_int_ = 0.0009). Also two variants in the proximity of *CYP1A1* (rs17861099, rs17861118) had potential interactions, with *P*_int_ = 0.001. Both variants are in high LD with each other (*r*^2^ = 0.83), but not with the variant rs181042206 identified in the association analysis for percent mammographic density (*r*^2^ <0.20).

**Table 3:** Association between mammographic density and current use of menopausal hormone therapy stratified by genotypes of ten SNPs showing the lowest *P* values for interaction

SNP	SNP type	Chr	Gene	Density measure	Homozygous reference genotype (N)		Heterozygous genotype (N)		Homozygous non-reference genotype (N)		*P* interaction
					Beta^a^ (95 % CI)	*P*	Beta^a^ (95 % CI)	*P*	Beta^a^ (95 % CI)	*P*	
					T/T (2000)		T/G (3167)		G/G (1131)		
rs9358531	Imputed	6	6.5 kb 5′ of PRL	PD	0.23 (0.08, 0.38)	2.5 × 10^−03^	0.46 (0.34, 0.58)	5.1 × 10^−14^	0.69 (0.49, 0.89)	1.4 × 10^−11^	0.0004
				DA	0.38 (0.18, 0.58)	2.1 × 10^−04^	0.6 (0.44, 0.76)	1.2 × 10^−13^	0.85 (0.59, 1.12)	2.7 × 10^−10^	0.008
				NDA	−0.17 (−0.39, 0.05)	1.4 × 10^−01^	−0.29 (−0.47, −0.12)	1.2 × 10^−03^	−0.58 (−0.87, −0.29)	1.0 × 10^−04^	0.02
					G/G (2138)		G/A (3142)		A/A (1018)		
rs9356811	Imputed	6	5.2 kb 5′ of PRL	PD	0.24 (0.1, 0.39)	1.1 × 10^−03^	0.48 (0.36, 0.6)	1.3 × 10^−14^	0.69 (0.48, 0.9)	1.5 × 10^−10^	0.0004
				DA	0.39 (0.2, 0.59)	6.6 × 10^−05^	0.6 (0.44, 0.76)	1.9 × 10^−13^	0.89 (0.61, 1.17)	4.6 × 10^−10^	0.005
				NDA	−0.17 (−0.38, 0.05)	1.2 × 10^−01^	−0.33 (−0.51, −0.15)	2.5 × 10^−04^	−0.51 (−0.82, −0.2)	1.1 × 10^−03^	0.05
					C/C (2158)		C/T (3124)		T/T (1016)		
rs10946546	Imputed	6	17 kb 5′ of PRL	PD	0.22 (0.07, 0.37)	3.2 × 10^−03^	0.51 (0.39, 0.63)	2.2 × 10^−16^	0.63 (0.42, 0.84)	3.0 × 10^−09^	0.0004
				DA	0.39 (0.19, 0.58)	9.0 × 10^−05^	0.64 (0.48, 0.8)	4.8 × 10^−15^	0.77 (0.49, 1.05)	4.9 × 10^−08^	0.02
				NDA	−0.11 (−0.32, 0.1)	3.1 × 10^−01^	−0.37 (−0.55, −0.2)	3.4 × 10^−05^	−0.49 (−0.8, −0.19)	1.5 × 10^−03^	0.01
					T/T (2230)		T/C (3093)		C/C (975)		
rs9393273	Imputed	6	8.1 kb 5′ of PRL	PD	0.24 (0.1, 0.39)	7.8 × 10^−04^	0.49 (0.37, 0.61)	4.8 × 10^−15^	0.68 (0.47, 0.89)	5.0 × 10^−10^	0.0005
				DA	0.4 (0.21, 0.59)	3.6 × 10^−05^	0.62 (0.46, 0.78)	8.5 × 10^−14^	0.86 (0.58, 1.15)	2.4 × 10^−09^	0.01
				NDA	−0.16 (−0.37, 0.05)	1.3 × 10^−01^	−0.33 (−0.51, −0.16)	2.4 × 10^−04^	−0.54 (−0.85, −0.23)	6.9 × 10^−04^	0.03
					A/A (2079)		A/G (3154)		G/G (1065)		
rs12525289	Imputed	6	9.9 kb 5′ of PRL	PD	0.24 (0.09, 0.39)	1.5 × 10^−03^	0.47 (0.35, 0.59)	2.0 × 10^−14^	0.68 (0.47, 0.88)	7.8 × 10^−11^	0.0005
				DA	0.4 (0.2, 0.59)	7.3 × 10^−05^	0.59 (0.43, 0.76)	4.3 × 10^−13^	0.88 (0.61, 1.15)	2.1 × 10^−10^	0.009
				NDA	−0.14 (−0.35, 0.08)	2.1 × 10^−01^	−0.34 (−0.51, −0.16)	1.9 × 10^−04^	−0.53 (−0.82, −0.23)	5.4 × 10^−04^	0.03
					G/G (2090)		G/A (3142)		A/A (1066)		
rs12199382	Imputed	6	24 kb 5′ of PRL	PD	0.21 (0.07, 0.36)	4.5 × 10^−03^	0.51 (0.39, 0.63)	1.8 × 10^−16^	0.62 (0.41, 0.82)	2.8 × 10^−09^	0.0006
				DA	0.37 (0.17, 0.56)	2.5 × 10^−04^	0.65 (0.49, 0.81)	1.7 × 10^−15^	0.76 (0.49, 1.03)	3.5 × 10^−08^	0.02
				NDA	−0.13 (−0.35, 0.08)	2.2 × 10^−01^	−0.35 (−0.53, −0.17)	1.1 × 10^−04^	−0.49 (−0.79, −0.2)	1.1 × 10^−03^	0.02
					G/G (2629)		G/A (2873)		A/A (796)		
rs9542456	Genotyped	13	505 kb 3′ of ATXN8OS	PD	0.59 (0.46, 0.73)	4.7 × 10^−18^	0.33 (0.2, 0.45)	4.1 × 10^−07^	0.22 (−0.01, 0.45)	5.9 × 10^−02^	0.0009
				DA	0.75 (0.57, 0.93)	2.0 × 10^−16^	0.45 (0.28, 0.61)	1.8 × 10^−07^	0.44 (0.14, 0.75)	4.7 × 10^−03^	0.02
				NDA	−0.43 (−0.63, −0.24)	1.6 × 10^−05^	−0.27 (−0.45, −0.08)	4.7 × 10^−03^	0.02 (−0.32, 0.36)	9.2 × 10^−01^	0.02
					T/T (2241)		T/G (3092)		G/G (965)		
rs12524161	Imputed	6	46 kb 5′ of PRL	PD	0.26 (0.12, 0.41)	2.8 × 10^−04^	0.47 (0.35, 0.6)	3.9 × 10^−14^	0.68 (0.47, 0.89)	4.6 × 10^−10^	0.001
				DA	0.42 (0.23, 0.61)	1.2 × 10^−05^	0.6 (0.44, 0.76)	4.8 × 10^−13^	0.86 (0.58, 1.14)	2.6 × 10^−09^	0.01
				NDA	−0.14 (−0.35, 0.06)	1.7 × 10^−01^	−0.34 (−0.51, −0.16)	2.3 × 10^−04^	−0.57 (−0.88, −0.26)	3.3 × 10^−04^	0.02
					C/C (5358)		C/T (899)		T/T (41)		
rs17861099	Imputed	15	462 bp 5′ of CYP1A1	PD	0.48 (0.39, 0.58)	2.4 × 10^−21^	0.09 (−0.14, 0.32)	4.3 × 10^−01^	−0.22 (−1.8, 1.37)	7.9 × 10^−01^	0.001
				DA	0.65 (0.52, 0.78)	1.1 × 10^−21^	0.13 (−0.17, 0.43)	3.9 × 10^−01^	0.01 (−2.09, 2.1)	9.9 × 10^−01^	0.002
				NDA	−0.35 (−0.5, −0.21)	1.8 × 10^−06^	0.02 (−0.32, 0.35)	9.2 × 10^−01^	−0.18 (−2.48, 2.13)	8.8 × 10^−01^	0.04
					G/G (5337)		G/A (923)		A/A (38)		
rs17861118	Imputed	15	8.3 kb 5′ of CYP1A1	PD	0.49 (0.39, 0.59)	2.6 × 10^−21^	0.1 (−0.12, 0.33)	3.8 × 10^−01^	−0.23 (−1.81, 1.35)	7.8 × 10^−01^	0.001
				DA	0.66 (0.52, 0.79)	3.4 × 10^−22^	0.09 (−0.21, 0.39)	5.6 × 10^−01^	−0.02 (−2.11, 2.08)	9.9 × 10^−01^	0.0006
				NDA	−0.34 (−0.49, −0.19)	5.0 × 10^−06^	−0.08 (−0.41, 0.24)	6.1 × 10^−01^	−0.18 (−2.48, 2.13)	8.8 × 10^−01^	0.08

^a^Adjusted for study, reference age, case status, former use of menopausal hormone therapy, body mass index, number of pregnancies and principal components. *SNP* single nucleotide polymorphism, *Chr* chromosome, *PD* percent density (%), square-root-transformed, *DA* dense area (cm^2^), square-root-transformed, *NDA* non-dense area (cm^2^), square-root-transformed

The ten SNPs showing the lowest *P*_int_ for percent mammographic density in this study did not overlap with the 14 SNPs recently identified to be potential modifiers of overall/lobular breast cancer risk associated with MHT use [29]. These 14 previously identified SNPs also did not show clear evidence of interaction with current use of MHT on percent mammographic density when analyzing the whole study sample (Additional file 5: Table S4). However, when testing for three-way interactions between SNP, current MHT use and case status, three SNPs in introns of *PLCG2* on chromosome 16 (rs7192724, rs4888190, rs17202296) had differential interaction effects for cases and non-cases (*P*_int_ SNP × MHT × case status = 0.005, 0.007, and 0.019, respectively, Additional file 6: Table S5). Potential interactions with current use of MHT on percent density were observed solely for cases (*P*_int_ = 0.001, 0.0004, 0.001, respectively), but not in non-cases (*P*_int_ = 0.57, 0.86, 0.81, respectively).

In our study sample, the ten variants known from genome-wide association studies (GWASs) were associated with percent mammographic density, dense area or non-dense area as expected, also due to overlap between the discovery samples and the studies included in this work (Additional file 7: Table S7). None of them showed a significant interaction with current use of MHT on percent mammographic density (Additional file 8: Table S8).

## Discussion

We assessed whether 3,878 SNPs selected based on a meta-analysis of four genome-wide case-only gene-environment interaction studies, candidate gene studies and GWAS of percent mammographic density are differentially associated with mammographic density according to current use of MHT. After accounting for multiple testing, there were no significant interactions for mammographic density between the investigated SNPs and current use of MHT. However, three SNPs in *PLCG2* showing potential interaction with MHT use for overall breast cancer risk in our previous study also showed potential interactions with MHT use for mammographic density in this study, but solely among cases. Thus, the results of this study may indicate a potential common pathway between the biological mechanisms underlying higher mammographic density and increased risk of breast cancer associated with MHT use.

The variants rs7192724, rs17202296, and rs4888190 are located within *PLCG2,* although in two different intronic regions. All three SNPs are located within 6 kb and are in relatively strong LD (D’ >0.85, *R*^2^ >0.71). As none of them are in LD with a coding variant, the effect of the causal SNP may be exerted through a regulatory mechanism. Using HaploReg and the UCSC genome browser, we identified rs12448089 in strong LD with rs4888190 (D’ = 0.98, *R*^2^ = 0.87) as a potential functional variant. SNP rs12448089 is located in a DNase I hypersensitive site and binding motifs of several transcription factors (Additional file 9: Figure S1). Genotypes for rs12448089 were not available in this study.

*PLCG2* encodes phospholipase C-gamma 2, an enzyme involved in the transmission of activation signals across the cell membranes predominantly of B cells [44] as well as natural killer cells [45]. PLCG2 plays an important role in immune response regulation [46] and aberrant functioning of PLCG2 due to exon deletions [47] or a missense mutation [46] causes autoimmunity diseases. With regard to breast cancer, *PLCG2* has been identified as an irradiation-responsive gene and a potential modifier of breast cancer risk in *BRCA2-*mutation carriers [48]. The functional role of PLCG2 in breast cancer has to be further elucidated before it is possible to derive a model that would explain our findings with *PLCG2* variants.

Two studies reported significant interactions between MHT use and genetic variants in hormone-related genes on mammographic density [24, 28]. The first study of 232 postmenopausal women in clinical trials of estrogen therapy and combined estrogen-progesterone therapy investigated the change in percent mammographic density between a baseline mammogram and a mammogram taken at about one year after randomization [24]. Significant interactions between the assignment to the estrogen therapy/combined estrogen-progesterone therapy treatment arm and the *CYP1B1* Val432Leu (rs1056836) polymorphism and the *AKR1C4* Leu311Val (rs17134592) polymorphism were reported (*P*_int_ = 0.0004 and 0.001, respectively). The *CYP1B1* Val432Leu polymorphism was previously evaluated for interaction with MHT use in 538 women and no interaction for percent mammographic density was found (*P*_int_ = 0.70) [22]. In this study, neither the *CYP1B1* Val432Leu nor the *AKR1C4* Leu311Val polymorphism showed an interaction with current use of MHT on percent mammographic density (*P*_int_ = 0.19 and 0.47, respectively). The second and larger study of 2,036 postmenopausal women who attended the Norwegian Breast Cancer Screening Program reported a significant interaction between SNP rs10946545 located 1.4kb 3′ of *PRL* and current use of estrogen-progesterone therapy for percent mammographic density (*P*_int_ = 0.0008) [28]. This SNP did not show a potential interaction with current MHT use here (*P*_int_ = 0.07), however, the non-significant interaction was in the same direction as that previously reported [28]. Several other variants located in the 5′ region of *PRL* had the lowest *P*_int_ values (Table 3), however, they were not in LD with rs10946545. The function of those SNPs near *PRL* is unknown, the SNP with the strongest interaction (rs9358531) is not located in an obvious regulatory element (Additional file 10: Figure S2). *PRL* encoding the hormone prolactin may be an interesting candidate for further studies of interactions between genetic variants and use of MHT with respect to mammographic density. In humans, prolactin is primarily produced in the anterior pituitary gland, but it is also expressed in the mammary gland itself and adipose tissue, among others [49]. In postmenopausal women, higher prolactin levels have been associated with higher mammographic density in three studies [50–52], two other studies found no association [53, 54].

Ten loci have been identified by GWAS to be associated with percent mammographic density, *ZNF365*-rs10995190 [10, 30], 12q24-rs1265507 [31], and *AREG*-rs10034692, *PRDM6*-rs186749, *ESR1*-rs12665607, 8p11.23-rs7816345, *LSP1*-rs3817198, *IGF1*-rs703556, *TMEM184B*-rs7289126, and *SGSM3*-rs17001868 [10]. The *ZNF365, ESR1, LSP1* and *SGSM3* loci are also associated with breast cancer risk [10, 30], implicating a shared genetic basis of mammographic density and breast cancer. This is also supported by the findings of a study, which assessed a polygenic score based on mammographic density GWAS with respect to breast cancer risk [8]. Women in the top 10 % of the score had an associated 31 % increased risk of breast cancer compared to women in the bottom 10 % of the score. The locus on 12q24 on the other hand, seems not to be associated with breast cancer risk [31]. Our data indicated that associations of the SNPs identified by GWAS with mammographic density are unlikely to be modified by MHT use.

Unlike in previous studies investigating interactions between genetic variants and MHT use with regard to mammographic density, the comprehensive set of SNPs investigated here was not only based on candidate genes, but selected also based on genome-wide case-only gene-environment interaction studies on breast cancer risk. GWAS have been generally more successful at identifying novel associations with complex diseases than candidate gene or linkage studies [55]. Thus, this approach to selecting SNPs can be considered as more promising. On the other hand, in doing so we made the fairly strong assumption that the biological mechanisms involved in the increase in breast cancer risk associated with MHT use at least in part overlap with the mechanisms involved in the increase in mammographic density associated with MHT use. This does not necessarily have to be true, although we identified polymorphisms in *PLCG2* that had interactions with MHT use for both breast cancer risk and mammographic density.

We were able to account for potential confounders of the association between current use of MHT and mammographic density such as age, BMI, case status, and number of pregnancies. All included studies were genotyped using the same genotyping platform. The study design and methods used to measure mammographic density varied between studies included in this analysis. However, the estimates for association and interaction were consistent across studies, supporting the robustness of our results. The sample size of our study was fairly large and the power was sufficient to detect strong SNP-MHT interactions on mammographic density with a beta value of 0.40. However, the power to detect weak to moderate interactions, in the range we observed in this dataset with betas of about 0.20, was limited and would require a sample at least four times larger. It is therefore possible that we missed potentially relevant interactions. Another limitation is the lack of type-specific information on MHT use. We could therefore not investigate interactions with use of combined estrogen-progesterone therapies, which seem to be more strongly associated with an increased mammographic density than estrogen-only therapies [16, 17]. Studies with type-specific information on MHT use could therefore also observe interactions of larger magnitudes compared to the magnitudes of the interactions observed here. However, the four genome-wide gene-environment interaction studies on which we partly based our SNP selection also investigated solely interactions with use of any MHT.

## Conclusion

We observed little evidence for strong interactions between the investigated SNPs and MHT use on mammographic density. The study identified variants near *PRL* and also in *PLCG2* for cases only*,* as potential modifiers of the association between MHT use and mammographic density. These findings will need to be confirmed in larger independent studies. The identification of additional gene-MHT interactions is likely to require very large (genome-wide) studies with type-specific information on MHT use, given the likely moderate/weak magnitude of such interactions.

## Additional files

Additional file 1: Table S1. Description of studies. (DOC 84 kb) Additional file 2: Table S2. Selected characteristics of the study population by study. *ABCFS* Australian breast cancer family study, *BBCC* Bavarian breast cancer cases and controls, *MCBS* Mayo clinic breast cancer study, *MCCS* Melbourne collaborative cohort study, *MEC* Multi-ethnic cohort, *MMHS* Mayo mammography health study, *OFBCR* Ontario familial breast cancer registry, *SASBAC* Singapore and Sweden breast cancer study, *EPIC* European prospective investigation into cancer and nutrition, *SIBS* Sisters in breast screening study. (DOC 87 kb) Additional file 3: Table S3. Ten single nucleotide polymorphisms (*SNPs*) showing lowest *P* values for interaction with current use of menopausal hormone therapy (*MHT*) on percent mammographic density. *Chr* chromosome. (DOC 48 kb) Additional file 4: Table S6.
**a** Beta estimates for interaction between single nucleotide polymorphisms (*SNPs*) and use of hormone replacement therapy (*MHT*) on percent density and for association between SNP and percent density. **b** beta estimates for interaction between SNPs and use of MHT on dense area (cm^2^) and for association between SNPs and dense area (cm^2^). **c** beta estimates for interaction between SNPs and use of MHT on non-dense area (cm^2^) and for association between SNPs and non-dense area (cm^2^). (ZIP 2747 kb) Additional file 5: Table S4. Beta estimates and confidence intervals for interaction between selected single nucleotide polymorphisms (*SNPs*) and current menopausal hormone therapy (*MHT*) use on square-root-transformed percent mammographic density. (DOC 65 kb) Additional file 6: Table S5. Beta estimates for interactions between selected single nucleotide polymorphisms (*SNPs*) in *PLCG2* and current use of menopausal hormone therapy (*MHT*) on square-root-transformed percent mammographic density in women not diagnosed with breast cancer and in women with breast cancer. *Chr* chromosome. (DOC 37 kb) Additional file 7: Table S7. Estimates for association between single nucleotide polymorphisms (*SNPs*) identified in genome-wide association studies (*GWAS*) and percent density, dense area and non-dense area. *Chr* chromosome. (DOC 46 kb) Additional file 8: Table S8. Estimates for interaction between use of menopausal hormone therapy and single nucleotide polymorphisms (*SNPs*) identified in genome-wide association studies (*GWAS*) for percent density, dense area and non-dense area. *Chr* chromosome. (DOC 46 kb) Additional file 9: Figure S1. University of California Santa Cruz (*UCSC*) Genome browser view (chr16:81958200–81963700) showing the intergenic position of single nucleotide polymorphisms (*SNPs*) rs7192724, rs17202296, and rs4888190, and correlated SNPs (r2>0.6 in 1000 Genomes CEU Pilot population). (DOC 231 kb) Additional file 10: Figure S2. University of California Santa Cruz (*UCSC*) Genome browser view (chr6:22308200–22348900) showing the position of single nucleotide polymorphisms (*SNPs*) rs9356811, rs10946546, rs9358531, rs9393273, rs12525289, rs12199382 and rs12524161. (DOC 261 kb)

## Abbreviations

ABCFS: Australian breast cancer family study
BBCC: Bavarian breast cancer cases and controls
BCAC: Breast Cancer Association Consortium
BMI: body mass index
chr: chromosome
CI: confidence interval
EPIC: European prospective investigation into cancer and nutrition
FDR: false discovery rate
GWAS: genome-wide association studies
iCOGS: custom Illumina iSelect genotyping array
LD: linkage disequilibrium
MAF: MCBS, Mayo clinic breast cancer study
MCCS: Melbourne collaborative cohort study
MEC: Multi-ethnic cohort, minor allele frequency
MHT: menopausal hormone therapy
MMHS: Mayo mammography health study
OFBCR: Ontario familial breast cancer registry
*P*_int_: *P*-value for interaction
SASBAC: Singapore and Sweden breast cancer study
SIBS: Sisters in breast screening study
SNP: single nucleotide polymorphism
UCSC: University of California Santa Cruz
