# Supplementary material for: A comprehensive evaluation of interaction between genetic variants and use of menopausal hormone therapy on mammographic density
Source: Breast Cancer Res. 2015 Aug 16;17(1):110. doi: 10.1186/s13058-015-0625-9 (PMC4537547; doi:10.1186/s13058-015-0625-9)
Supplement: Additional file 1: Table S1. — Description of studies. (DOC 84 kb) [file 13058_2015_625_MOESM1_ESM.doc]

**Supplementary Table 1.** Description of studies.

| **Study Acronym** | **Study Name**  **[Reference]** | **Study design** | **Recruitment base** | |
| --- | --- | --- | --- | --- |
|  |  |  | **Cases** | **Non-cases** |
| ABCFS | Australian Breast Cancer Family Study [1] | Case-control | All cases diagnosed age <40 plus a random sample of those diagnosed ages 40-59 from cancer registries in Victoria and New South Wales, plus a limited number diagnosed aged 60-69; cases living in Melbourne recruited from 1992-1999 and in Sydney from 1993-1998. | Identified from the electoral rolls in Melbourne from 1992-1998 and Sydney from 1993-1999. Frequency matched to cases by age in 5 year categories. |
| BBCC | Bavarian Breast Cancer Cases and Controls [2, 3] | Case-control | Consecutive, unselected cases with invasive breast cancer recruited at the University Breast Centre, Franconia in Northern Bavaria during 2002-2010. | Healthy women with no diagnosis of cancer aged 55 or older. Invited by a newspaper advertisement in Northern Bavaria, and recruited during 2002-2010. |
| EPIC | The European Prospective Investigation into Cancer and Nutrition [4] | Cohort | Women of European ancestry from the EPIC-Norfolk cohort, recruited in Norfolk, UK between 1993-97 and aged 39-79 at recruitment, who had been diagnosed with breast cancer between recruitment and the start of this study. Mammographic studies were undertaken as part of the UK National Health Service Breast Screening Program for women aged between 50 and 64 years. | Women of European ancestry from the EPIC-Norfolk cohort, recruited in Norfolk, UK between 1993-97 and aged 39-79 at recruitment, who had not been diagnosed with breast cancer by the start of this study. Mammographic studies were undertaken as part of the UK National Health Service Breast Screening Program for women aged between 50 and 64 years. |
| MCBCS | Mayo Clinic Breast Cancer Study [5] | Case-control | Incident cases residing in 6 states (MN, WI, IA, IL, ND, SD) seen at the Mayo Clinic in Rochester, MN from 2002-2010. | Women presenting for general medical examination at the Mayo Clinic from 2002-2010; frequency matched to cases on age, ethnicity and county/state. |
| MCCS | Melbourne Collaborative Cohort Study [6] | Nested case-control | Incident cases from the cohort of 24,469 women, diagnosed during the follow-up from baseline (1990-1994) to 2008. | Random sample of the initial cohort. |
| MEC | Multi-Ethnic Cohort [7] | Nested case-control | Incident cases identified from SEER cancer registries in Los Angeles County & State registries in California & Hawaii, USA from 1993-2002. Grouped by self-reported ethnicity. | Women without cancer from the same States, recruited concurrently with cases & frequency matched to cases by age at blood-draw & self-reported ethnicity. |
| MMHS | Mayo Mammography Health Study [8] | Nested case-control | Incident cases from the cohort of 19,924 participating women scheduled for a screening mammogram from 2003-2006 at the Mayo Clinic in Rochester, MN. Women were invited to take part if they were at least 35 years old, residents of MN, IA, or WI (tri-state), and had no personal history of breast cancer. | A nested case-control design was used to minimize the number of participants for genotyping. Controls were matched to cases on age, menopausal status, year of exam, and state of residence. |
| OFBCR | Ontario Familial Breast Cancer Registry [9] | Case-control with increased familial risk | Invasive cases (all aged 20-54 years and a random sample aged 55-69 years) were identified from the Ontario Cancer Registry 1996-1998. All those at high genetic risk were eligible. Random samples of women not meeting these criteria were also asked to participate. During 2001-2005, some enrolment continued, but was limited to minority and high-risk families. | Identified by calling randomly selected residential telephone numbers in the same geographical region from 1998-2001; frequency matched to cases by age in 5 year categories. |
| SASBAC | Singapore and Sweden Breast Cancer Study [10] | Case-control | Women diagnosed in Sweden aged 50-74 in 1993-1995. | Population-based controls frequency matched by age to the cases. |
| SIBS | The Sisters in Breast Screening Study [11] | Family | No cases included. | Women of European ancestry recruited via the UK National Health Service Breast Screening Program for women aged between 50 and 64 years, with the requirement that they had a close female relative who was also part of the screening program. Women included in this study had no previous breast cancer. |

**Reference List**

1. Dite GS, Jenkins MA, Southey MC, Hocking JS, Giles GG, McCredie MR, Venter DJ, Hopper JL: **Familial risks, early-onset breast cancer, and BRCA1 and BRCA2 germline mutations**. *J Natl Cancer Inst* 2003, **95**(6):448-457.

2. Fasching PA, Loehberg CR, Strissel PL, Lux MP, Bani MR, Schrauder M, Geiler S, Ringleff K, Oeser S, Weihbrecht S *et al*: **Single nucleotide polymorphisms of the aromatase gene (CYP19A1), HER2/neu status, and prognosis in breast cancer patients**. *Breast Cancer Res Treat* 2008, **112**(1):89-98.

3. Schrauder M, Frank S, Strissel PL, Lux MP, Bani MR, Rauh C, Sieber CC, Heusinger K, Hartmann A, Schulz-Wendtland R *et al*: **Single nucleotide polymorphism D1853N of the ATM gene may alter the risk for breast cancer**. *J Cancer Res Clin Oncol* 2008, **134**(8):873-882.

4. Day N, Oakes S, Luben R, Khaw KT, Bingham S, Welch A, Wareham N: **EPIC-Norfolk: study design and characteristics of the cohort. European Prospective Investigation of Cancer**. *Br J Cancer* 1999, **80 Suppl 1**:95-103.

5. Olson JE, Ma CX, Pelleymounter LL, Schaid DJ, Pankratz VS, Vierkant RA, Fredericksen ZS, Ingle JN, Wu Y, Couch F *et al*: **A comprehensive examination of CYP19 variation and breast density**. *Cancer Epidemiol Biomarkers Prev* 2007, **16**(3):623-625.

6. Giles GG, English DR: **The Melbourne Collaborative Cohort Study**. *IARC Sci Publ* 2002, **156**:69-70.

7. Kolonel LN, Henderson BE, Hankin JH, Nomura AM, Wilkens LR, Pike MC, Stram DO, Monroe KR, Earle ME, Nagamine FS: **A multiethnic cohort in Hawaii and Los Angeles: baseline characteristics**. *Am J Epidemiol* 2000, **151**(4):346-357.

8. Vachon CM, Pankratz VS, Scott CG, Haeberle L, Ziv E, Jensen MR, Brandt KR, Whaley DH, Olson JE, Heusinger K *et al*: **The contributions of breast density and common genetic variation to breast cancer risk**. *J Natl Cancer Inst* 2015, **107**(5).

9. John EM, Hopper JL, Beck JC, Knight JA, Neuhausen SL, Senie RT, Ziogas A, Andrulis IL, Anton-Culver H, Boyd N *et al*: **The Breast Cancer Family Registry: an infrastructure for cooperative multinational, interdisciplinary and translational studies of the genetic epidemiology of breast cancer**. *Breast Cancer Res* 2004, **6**(4):R375-389.

10. Wedren S, Lovmar L, Humphreys K, Magnusson C, Melhus H, Syvanen AC, Kindmark A, Landegren U, Fermer ML, Stiger F *et al*: **Oestrogen receptor alpha gene haplotype and postmenopausal breast cancer risk: a case control study**. *Breast Cancer Res* 2004, **6**(4):R437-449.

11. Varghese JS, Smith PL, Folkerd E, Brown J, Leyland J, Audley T, Warren RM, Dowsett M, Easton DF, Thompson DJ: **The heritability of mammographic breast density and circulating sex-hormone levels: two independent breast cancer risk factors**. *Cancer Epidemiol Biomarkers Prev* 2012, **21**(12):2167-2175.
