# Supplementary material for: A comprehensive evaluation of interaction between genetic variants and use of menopausal hormone therapy on mammographic density
Source: Breast Cancer Res. 2015 Aug 16;17(1):110. doi: 10.1186/s13058-015-0625-9 (PMC4537547; doi:10.1186/s13058-015-0625-9)
Supplement: Additional file 2: Table S2. — Selected characteristics of the study population by study. ABCFS Australian breast cancer family study, BBCC Bavarian breast cancer cases and controls, MCBS Mayo clinic breast cancer study, MCCS Melbourne collaborative cohort study, MEC Multi-ethnic cohort, MMHS Mayo mammography health study, OFBCR Ontario familial breast cancer registry, SASBAC Singapore and Sweden breast cancer study, EPIC European prospective investigation into cancer and nutrition, SIBS Sisters in breast screening study. (DOC 87 kb) [file 13058_2015_625_MOESM2_ESM.doc]

**Supplementary Table 2.** Selected characteristics of the study population by study.

|  | **ABCFS** | **BBCC** | | | **MCBCS** | | | **MCCS** |  |  | **MEC** |  |  |
| --- | --- | --- | --- | --- | --- | --- | --- | --- | --- | --- | --- | --- | --- |
| **Characteristic** | **Cases** | **Cases** | **Non-cases** | **Overall** | **Cases** | **Non-cases** | **Overall** | **Cases** | **Non-cases** | **Overall** | **Cases** | **Non-cases** | **Overall** |
|  |  |  |  |  |  |  |  |  |  |  |  |  |  |
| **Total number** | 30 | 386 | 240 | 626 | 441 | 417 | 858 | 50 | 25 | 75 | 100 | 72 | 172 |
|  |  |  |  |  |  |  |  |  |  |  |  |  |  |
| **Reference age, years** |  |  |  |  |  |  |  |  |  |  |  |  |  |
| mean | 44.9 | 65.7 | 62.1 | 64.3 | 62.7 | 62.9 | 62.8 | 62.0 | 59.3 | 61.1 | 61.8 | 59.0 | 60.6 |
| standard deviation | 6.3 | 8.6 | 7.3 | 8.3 | 9.5 | 9.6 | 9.5 | 6.1 | 7.0 | 6.5 | 8.0 | 8.1 | 8.2 |
|  |  |  |  |  |  |  |  |  |  |  |  |  |  |
| **BMI, kg/m^2^** |  |  |  |  |  |  |  |  |  |  |  |  |  |
| mean | 24.2 | 27.6 | 25.9 | 26.9 | 28.5 | 27.5 | 28.0 | 27.9 | 26.1 | 27.3 | 25.9 | 25.4 | 25.7 |
| standard deviation | 4.3 | 5.3 | 4.4 | 5.2 | 5.5 | 5.2 | 5.4 | 5.4 | 4.1 | 5.1 | 5.8 | 5.4 | 5.6 |
|  |  |  |  |  |  |  |  |  |  |  |  |  |  |
| **MHT use** |  |  |  |  |  |  |  |  |  |  |  |  |  |
| never | 23 (76.7) | 237 (61.4) | 108 (45.0) | 345 (55.1) | 157 (35.6) | 103 (24.7) | 260 (20.3) | 39 (78.0) | 19 (76.0) | 58 (77.3) | 19 (19.0) | 13 (18.1) | 32 (18.6) |
| former | 4 (13.3) | 95 (24.6)) | 94 (39.2) | 189 (30.2) | 264 (59.9) | 190 (45.6) | 454 (52.9) | 2 (4.0) | 5 (20.0) | 7 (9.3) | 20 (20.0) | 7 (9.7) | 27 (15.7) |
| current | 3 (10.0) | 54 (14.0) | 38 (15.8) | 92 (14.7) | 20 (4.5) | 124 (29.7) | 144 (16.8) | 9 (18.) | 1 (4.0) | 10 (13.4) | 61 (61.0) | 52 (72.2) | 113 (65.7) |
|  |  |  |  |  |  |  |  |  |  |  |  |  |  |
| ***Parity*** |  |  |  |  |  |  |  |  |  |  |  |  |  |
| nulliparous | 8 (26.7) | 52 (13.5) | 37 (15.4) | 89 (14.2) | 47 (10.7) | 60 (14.4) | 107 (12.5) | 4 (8.0) | 2 (8.0) | 6 (8.0) | 16 (16.0) | 19 (26.4) | 35 (20.4) |
| 1 full-term pregnancy | 0 (0.0) | 99 (25.6) | 58 (24.2) | 157 (25.1) | 40 (9.1) | 29 (7.0) | 69 (8.0) | 5 (10.0) | 1 (4.0) | 6 (8.0) | 11 (11.0) | 6 (8.3) | 17 (9.9) |
| 2 full-term pregnancies | 11 (36.7) | 149 (38.6) | 104 (43.3) | 253 (40.4) | 138 (31.3) | 110 (26.4) | 248 (28.9) | 15 (30.0) | 11 (44.0) | 26 (34.7) | 23 (23.0) | 22 (30.6) | 45 (26.2) |
| ≥ 3 full-term pregnancies | 11 (36.7) | 86 (22.3) | 41 (17.1) | 127 (20.3) | 216 (49.0) | 218 (52.3) | 434 (50.6) | 26 (52.0) | 11 (44.0) | 37 (49.3) | 50 (50.0) | 25 (34.7) | 75 (43.6) |
|  |  |  |  |  |  |  |  |  |  |  |  |  |  |

**Supplementary Table 2. (continued)**

|  | **MMHS** |  |  | **OFBCR** | **SASBAC** |  |  | **EPIC** |  |  | **SIBS** |
| --- | --- | --- | --- | --- | --- | --- | --- | --- | --- | --- | --- |
| **Characteristic** | **Cases** | **Non-cases** | **Overall** | **Cases** | **Cases** | **Non-cases** | **Overall** | **Cases** | **Non-cases** | **Overall** | **Non-cases** |
|  |  |  |  |  |  |  |  |  |  |  |  |
| **Total number** | 299 | 717 | 1016 | 67 | 804 | 676 | 1480 | 67 | 725 | 792 | 1182 |
|  |  |  |  |  |  |  |  |  |  |  |  |
| **Reference age, years** |  |  |  |  |  |  |  |  |  |  |  |
| mean | 65.0 | 63.9 | 64.2 | 55.3 | 62.9 | 63.0 | 62.9 | 67.3 | 68.1 | 68.0 | 60.6 |
| standard deviation | 8.3 | 9.4 | 9.1 | 6.8 | 6.2 | 6.2 | 6.2 | 6.3 | 5.1 | 5.2 | 5.8 |
|  |  |  |  |  |  |  |  |  |  |  |  |
| **BMI, kg/m^2^** |  |  |  |  |  |  |  |  |  |  |  |
| mean | 29.0 | 27.9 | 28.2 | 26.5 | 25.7 | 25.6 | 25.7 | 27.7 | 27.7 | 27.7 | 27.2 |
| standard deviation | 7.0 | 5.7 | 6.1 | 4.9 | 3.9 | 4.1 | 4.0 | 4.6 | 4.8 | 4.8 | 5.2 |
|  |  |  |  |  |  |  |  |  |  |  |  |
| **MHT use** |  |  |  |  |  |  |  |  |  |  |  |
| never | 100 (33.4) | 244 (34.0) | 344 (33.9) | 29 (43.3) | 384 (47.8) | 383 (56.7) | 767 (51.8) | 45 (67.2) | 452 (62.3) | 497 (62.8) | 530 (44.8) |
| former | 122 (40.8) | 261 (36.4) | 383 (37.7) | 37 (55.2) | 187 (23.3) | 194 (28.7) | 381 (25.7) | 9 (13.4) | 117 (16.1) | 126 (15.9) | 492 (41.6) |
| current | 77 (25.8) | 212 (29.6) | 289 (28.4) | 1 (1.5) | 233 (29.0) | 99 (14.6) | 332 (22.4) | 13 (19.4) | 156 (21.5) | 169 (21.3) | 160 (13.5) |
|  |  |  |  |  |  |  |  |  |  |  |  |
| ***Parity*** |  |  |  |  |  |  |  |  |  |  |  |
| nulliparous | 34 (11.4) | 76 (10.6) | 110 (10.8) | 14 (20.9) | 109 (13.6) | 55 (8.1) | 164 (11.1) | 12 (17.9) | 94 (13.0) | 106 (13.4) | 136 (11.5) |
| 1 full-term pregnancy | 21 (7.0) | 67 (9.3) | 88 (8.7) | 8 (12.0) | 171 (21.3) | 117 (17.3) | 288 (19.5) | 6 (9.0) | 78 (10.8) | 84 (10.6) | 131 (11.1) |
| 2 full-term pregnancies | 87 (29.1) | 215 (30.0) | 302 (29.7) | 18 (26.9) | 309 (38.4) | 252 (37.3) | 561 (37.9) | 25 (37.3) | 307 (42.3) | 332 (41.9) | 573 (48.5) |
| ≥ 3 full-term pregnancies | 157 (52.5) | 359 (50.1) | 516 (50.8) | 27 (40.3) | 215 (26.7) | 252 (37.3) | 467 (31.6) | 24 (35.8) | 246 (33.9) | 270 (34.1) | 342 (28.9) |
|  |  |  |  |  |  |  |  |  |  |  |  |
