# Supplementary material for: A comprehensive evaluation of interaction between genetic variants and use of menopausal hormone therapy on mammographic density
Source: Breast Cancer Res. 2015 Aug 16;17(1):110. doi: 10.1186/s13058-015-0625-9 (PMC4537547; doi:10.1186/s13058-015-0625-9)
Supplement: Additional file 3: Table S3. — Ten single nucleotide polymorphisms (SNPs) showing lowest P values for interaction with current use of menopausal hormone therapy (MHT) on percent mammographic density. Chr chromosome. (DOC 48 kb) [file 13058_2015_625_MOESM3_ESM.doc]

**Supplementary Table 3.** Ten SNPs showing lowest *P*-values for interaction with current use of MHT on percent mammographic density.

| **SNP** | **SNP type** | **Chr** | **Gene (RefSeq)** | **Percent density^a^** | | | **Dense area (cm^2^)^a^** | | **Non-dense area (cm^2^)^a^** | |
| --- | --- | --- | --- | --- | --- | --- | --- | --- | --- | --- |
|  |  |  |  | **Interaction beta^b^ (95% CI)** | ***P* inter-action** | **Adjusted *P* inter-action^c^** | **Interaction beta^b^ (95% CI)** | ***P* inter-action** | **Interaction beta^b^ (95% CI)** | ***P* inter-action** |
|  |  |  |  |  |  |  |  |  |  |  |
| rs9358531 | imputed | 6 | 6.5kb 5' of PRL | 0.24 (0.11, 0.37) | 0.0004 | 1.45 | 0.24 (0.06, 0.41) | 0.008 | -0.22 (-0.41, -0.03) | 0.02 |
| rs9356811 | imputed | 6 | 5.2kb 5' of PRL | 0.24 (0.11, 0.37) | 0.0004 | 0.73 | 0.25 (0.08, 0.43) | 0.005 | -0.2 (-0.39, 0) | 0.05 |
| rs10946546 | imputed | 6 | 17kb 5' of PRL | 0.23 (0.1, 0.36) | 0.0004 | 0.54 | 0.2 (0.03, 0.37) | 0.02 | -0.24 (-0.42, -0.05) | 0.01 |
| rs9393273 | imputed | 6 | 8.1kb 5' of PRL | 0.23 (0.1, 0.37) | 0.0005 | 0.50 | 0.23 (0.06, 0.41) | 0.01 | -0.22 (-0.41, -0.03) | 0.03 |
| rs12525289 | imputed | 6 | 9.9kb 5' of PRL | 0.23 (0.1, 0.35) | 0.0005 | 0.42 | 0.23 (0.06, 0.4) | 0.009 | -0.21 (-0.39, -0.02) | 0.03 |
| rs12199382 | imputed | 6 | 24kb 5' of PRL | 0.22 (0.1, 0.35) | 0.0006 | 0.39 | 0.21 (0.04, 0.38) | 0.02 | -0.21 (-0.4, -0.03) | 0.02 |
| rs9542456 | genotyped | 13 | 505kb 3' of ATXN8OS | -0.21 (-0.33, -0.09) | 0.0009 | 0.50 | -0.2 (-0.36, -0.03) | 0.02 | 0.21 (0.03, 0.39) | 0.02 |
| rs12524161 | imputed | 6 | 46kb 5' of PRL | 0.21 (0.08, 0.33) | 0.001 | 0.49 | 0.21 (0.05, 0.37) | 0.01 | -0.21 (-0.39, -0.03) | 0.02 |
| rs17861099 | imputed | 15 | 462bp 5' of CYP1A1 | -0.41 (-0.65, -0.16) | 0.001 | 0.45 | -0.52 (-0.84, -0.2) | 0.002 | 0.37 (0.02, 0.73) | 0.04 |
| rs17861118 | imputed | 15 | 8.3kb 5' of CYP1A1 | -0.42 (-0.67, -0.17) | 0.001 | 0.41 | -0.58 (-0.92, -0.25) | 0.0006 | 0.33 (-0.04, 0.7) | 0.08 |
|  |  |  |  |  |  |  |  |  |  |  |
| ^a^square-root transformed  ^b^adjusted for study, reference age, case status, former use of MHT, BMI, number of pregnancies and principal components  ^c^adjusted *P*-value for interaction, calculated by multiplying *P*-value by N tests (here N=3870) and dividing by assigned rank | | | | | | | | | | |
