# Supplementary material for: A comprehensive evaluation of interaction between genetic variants and use of menopausal hormone therapy on mammographic density
Source: Breast Cancer Res. 2015 Aug 16;17(1):110. doi: 10.1186/s13058-015-0625-9 (PMC4537547; doi:10.1186/s13058-015-0625-9)
Supplement: Additional file 5: Table S4. — Beta estimates and confidence intervals for interaction between selected single nucleotide polymorphisms (SNPs) and current menopausal hormone therapy (MHT) use on square-root-transformed percent mammographic density. (DOC 65 kb) [file 13058_2015_625_MOESM5_ESM.doc]

**Supplementary Table 4.** beta estimates and confidence intervals for interaction between selected SNPs and current menopausal hormone therapy use on square-root transformed percent mammographic density. The selected SNPs have previously been identified as potential modifiers of overall/lobular breast cancer risk associated with menopausal hormone therapy use .

| **SNP** | **Alleles** | **MAF** | **Chr** | **Position (hg19)** | **RefSeq Gene** | **Feature** | **Interaction beta^a^ (95% CI)** | ***P* inter-action** | ***P* interaction SNP×MHT×case-status** |
| --- | --- | --- | --- | --- | --- | --- | --- | --- | --- |
|  |  |  |  |  |  |  |  |  |  |
| rs7192724 | G>C | 23.2% | 16 | 81958298 | *PLCG2* | intronic | -0.11 (-0.25, 0.03) | 0.14 | 0.005 |
| rs4888190 | T>G | 25.5% | 16 | 81963618 | *PLCG2* | intronic | -0.16 (-0.29, -0.02) | 0.02 | 0.007 |
| rs17202296 | G>C | 25.5% | 16 | 81959191 | *PLCG2* | intronic | -0.15 (-0.28, -0.01) | 0.04 | 0.019 |
| rs7148646 | G>A | 28.2% | 14 | 37407036 | *SLC25A21* | intronic | -0.11 (-0.24, 0.02) | 0.09 | 0.070 |
| rs11080292 | G>A | 20.5% | 17 | 32998577 | 32kb 3' of *TMEM132E* |  | -0.10 (-0.25, 0.05) | 0.21 | 0.082 |
| rs9579199 | A>G | 27.6% | 13 | 29164783 | 68kb 5' of *POMP* | - | -0.02 (-0.14, 0.11) | 0.81 | 0.118 |
| rs9578047 | G>A | 27.6% | 13 | 29164731 | 68kb 5' of *POMP* | - | -0.02 (-0.14, 0.11) | 0.79 | 0.122 |
| rs848694 | G>A | 23.6% | 14 | 37371724 | *SLC25A21* | intronic | -0.06 (-0.21, 0.08) | 0.38 | 0.140 |
| rs16970162 | G>C | 20.3% | 17 | 32989786 | 23kb 3' of *TMEM132E* |  | -0.15 (-0.30, 0.00) | 0.05 | 0.300 |
| rs6506940 | A>G | 13.1% | 18 | 29333635 | 6kb 3' of *SLC25A52* |  | -0.21 (-0.39, -0.03) | 0.03 | 0.562 |
| rs11680872 | A>G | 25.3% | 2 | 192830249 | *TMEFF2* | intronic | -0.05 (-0.18, 0.09) | 0.50 | 0.661 |
| rs594334 | A>G | 11.3% | 18 | 29364523 | 24kb 5' of *SLC25A52* |  | -0.22 (-0.41, -0.02) | 0.03 | 0.676 |
| rs11654964 | A>C | 22.9% | 17 | 32989538 | 23kb 3' of *TMEM132E* |  | -0.13 (-0.28, 0.01) | 0.08 | 0.764 |
| rs7648642 | A>C | 46.9% | 3 | 119261375 | *CD80* | intronic | 0.07 (-0.05, 0.19) | 0.26 | 0.915 |
|  |  |  |  |  |  |  |  |  |  |
| ^a^adjusted for study, reference age, case status, former use of MHT, BMI, number of pregnancies and principal components  MAF: minor allele frequency; Chr: Chromosome; CI: confidence interval | | | | | | | | | |

**Reference**

1. Rudolph A, Hein R, Lindstrom S, Beckmann L, Behrens S, Liu J, Aschard H, Bolla MK, Wang J, Truong *T et* al**: Genetic modifiers of menopausal hormone replacement therapy and breast cancer risk: a genome-wide interaction stu**dy*. Endocrine-related cance*r 2013**,** 20(6):875-887.
