# Supplementary material for: A comprehensive evaluation of interaction between genetic variants and use of menopausal hormone therapy on mammographic density
Source: Breast Cancer Res. 2015 Aug 16;17(1):110. doi: 10.1186/s13058-015-0625-9 (PMC4537547; doi:10.1186/s13058-015-0625-9)
Supplement: Additional file 6: Table S5. — Beta estimates for interactions between selected single nucleotide polymorphisms (SNPs) in PLCG2 and current use of menopausal hormone therapy (MHT) on square-root-transformed percent mammographic density in women not diagnosed with breast cancer and in women with breast cancer. Chr chromosome. (DOC 37 kb) [file 13058_2015_625_MOESM6_ESM.doc]

**Supplementary Table 5.** beta estimates for interactions between selected SNPs in *PLCG2* and current use of MHT on square-root transformed percent mammographic density in women not diagnosed with breast-cancer and in breast cancer cases.

| **SNP** | **Chr** | **Position (hg19)** | **Gene (RefSeq)** | **Feature** | **Non-cases (N = 4054)** | | **Cases (N = 2244)** | | ***P* interaction**  **SNP×MHT×case-status** |
| --- | --- | --- | --- | --- | --- | --- | --- | --- | --- |
|  |  |  |  |  | **Interaction beta^a^ (95% CI)** | ***P* inter-action** | **Interaction beta^a^ (95% CI** | ***P* inter-action** |  |
|  |  |  |  |  |  |  |  |  |  |
| rs7192724 | 16 | 81958298 | *PLCG2* | intronic | 0.05 (-0.12, 0.22) | 0.57 | -0.39 (-0.63, -0.16) | 0.001 | 0.005 |
| rs4888190 | 16 | 81963618 | *PLCG2* | intronic | -0.01 (-0.18, 0.15) | 0.86 | -0.41 (-0.64, -0.19) | 0.0004 | 0.007 |
| rs17202296 | 16 | 81959191 | *PLCG2* | intronic | -0.02 (-0.19, 0.15) | 0.81 | -0.39 (-0.62, -0.16) | 0.001 | 0.019 |
|  |  |  |  |  |  |  |  |  |  |
| ^a^adjusted for study, reference age, former use of menopausal hormone therapy, BMI, number of pregnancies and principal components | | | | | | | | | |
