# Supplementary material for: A comprehensive evaluation of interaction between genetic variants and use of menopausal hormone therapy on mammographic density
Source: Breast Cancer Res. 2015 Aug 16;17(1):110. doi: 10.1186/s13058-015-0625-9 (PMC4537547; doi:10.1186/s13058-015-0625-9)
Supplement: Additional file 7: Table S7. — Estimates for association between single nucleotide polymorphisms (SNPs) identified in genome-wide association studies (GWAS) and percent density, dense area and non-dense area. Chr chromosome. (DOC 46 kb) [file 13058_2015_625_MOESM7_ESM.doc]

**Supplementary Table 7.** Estimates for association between SNPs identified in GWASs and percent density, dense area and non-dense area.

| **SNP** | **SNP type** | **Chr** | **Gene (RefSeq)** | **Percent density^a^** | | **Dense area (cm^2^)^a^** | | **Non-dense area (cm^2^)^a^** | |
| --- | --- | --- | --- | --- | --- | --- | --- | --- | --- |
|  |  |  |  | **beta^b^ (95% CI)** | ***P* value** | **beta^b^ (95% CI)** | ***P* value** | **beta^b^ (95% CI)** | ***P* value** |
|  |  |  |  |  |  |  |  |  |  |
| rs10034692 | genotyped | 4 | 61kb 5' of AREG | -0.07 (-0.12, -0.01) | 0.02 | -0.13 (-0.20, -0.06) | 0.0004 | -0.03 (-0.11, 0.05) | 0.46 |
| rs186749 | imputed | 5 | PRDM6 | -0.10 (-0.15, -0.05) | 7.9×10^-5^ | -0.12 (-0.19, -0.05) | 0.0005 | 0.11 (0.03, 0.18) | 0.005 |
| rs12665607 | genotyped | 6 | 4.3kb 3' of C6orf97 | 0.16 (0.07, 0.25) | 6.1×10^-4^ | 0.27 (0.15, 0.39) | 1.4×10^-5^ | 0.03 (-0.10, 0.17) | 0.61 |
| rs10995190 | genotyped | 10 | ZNF365 | -0.11 (-0.18, -0.05) | 0.001 | -0.24 (-0.33, -0.14) | 3.6×10^-7^ | -0.10 (-0.20, 0.00) | 0.05 |
| rs7816345 | genotyped | 8 | 52kb 3' of KCNU1 | 0.12 (0.05, 0.18) | 0.0003 | 0.07 (-0.02, 0.16) | 0.11 | -0.32 (-0.41, -0.22) | 3.9×10^-11^ |
| rs3817198 | genotyped | 11 | LSP1 | 0.07 (0.02, 0.13) | 0.006 | 0.13 (0.06, 0.20) | 0.0002 | 0.00 (-0.08, 0.08) | 0.99 |
| rs703556 | genotyped | 12 | 138kb 5' of IGF1 | -0.27 (-0.44, -0.11) | 0.001 | -0.42 (-0.64, -0.20) | 0.0002 | -0.09 (-0.33, 0.16) | 0.49 |
| rs1265507 | genotyped | 12 | 18kb 3' of LOC255480 | 0.10 (0.05, 0.15) | 4.2×10^-5^ | 0.13 (0.06, 0.19) | 0.0001 | -0.04 (-0.11, 0.03) | 0.24 |
| rs7289126 | genotyped | 22 | TMEM184B | -0.08 (-0.13, -0.03) | 0.002 | -0.08 (-0.15, -0.02) | 0.01 | 0.10 (0.03, 0.17) | 0.006 |
| rs17001868 | genotyped | 22 | SGSM3 | -0.09 (-0.15, -0.02) | 0.02 | -0.19 (-0.28, -0.10) | 5.7×10^-5^ | -0.19 (-0.29, -0.09) | 0.0002 |
|  |  |  |  |  |  |  |  |  |  |
| ^a^square-root transformed  ^b^adjusted for study, reference age, case status, former use of MHT, BMI, number of pregnancies and principal components | | | | | | | | | |
