# Supplementary material for: A comprehensive evaluation of interaction between genetic variants and use of menopausal hormone therapy on mammographic density
Source: Breast Cancer Res. 2015 Aug 16;17(1):110. doi: 10.1186/s13058-015-0625-9 (PMC4537547; doi:10.1186/s13058-015-0625-9)
Supplement: Additional file 8: Table S8. — Estimates for interaction between use of menopausal hormone therapy and single nucleotide polymorphisms (SNPs) identified in genome-wide association studies (GWAS) for percent density, dense area and non-dense area. Chr chromosome. (DOC 46 kb) [file 13058_2015_625_MOESM8_ESM.doc]

**Supplementary Table 8.** Estimates for interaction between use of menopausal hormone therapy and SNPs identified in GWASs for percent density, dense area and non-dense area.

| **SNP** | **SNP type** | **Chr** | **Gene (RefSeq)** | **Percent density^a^** | | **Dense area (cm^2^)^a^** | | **Non-dense area (cm^2^)^a^** | |
| --- | --- | --- | --- | --- | --- | --- | --- | --- | --- |
|  |  |  |  | **Interaction beta^b^ (95% CI)** | ***P* inter-action** | **Interaction beta^b^ (95% CI)** | ***P* inter-action** | **Interaction beta^b^ (95% CI)** | ***P* inter-action** |
|  |  |  |  |  |  |  |  |  |  |
| rs10034692 | genotyped | 4 | 61kb 5' of AREG | 0.07 (-0.06, 0.20) | 0.31 | 0.16 (-0.02, 0.33) | 0.08 | 0.21 (0.02, 0.40) | 0.03 |
| rs186749 | imputed | 5 | PRDM6 | -0.15 (-0.27, -0.02) | 0.02 | -0.17 (-0.33, 0.00) | 0.05 | 0.15 (-0.03, 0.34) | 0.11 |
| rs12665607 | genotyped | 6 | 4.3kb 3' of C6orf97 | 0.17 (-0.05, 0.39) | 0.14 | 0.25 (-0.05, 0.54) | 0.10 | -0.17 (-0.49, 0.16) | 0.31 |
| rs10995190 | genotyped | 10 | ZNF365 | 0.19 (0.02, 0.35) | 0.03 | 0.23 (0.01, 0.45) | 0.04 | -0.27 (-0.51, -0.02) | 0.03 |
| rs7816345 | genotyped | 8 | 52kb 3' of KCNU1 | -0.02 (-0.18, 0.14) | 0.77 | -0.01 (-0.23, 0.20) | 0.90 | 0.06 (-0.17, 0.30) | 0.59 |
| rs3817198 | genotyped | 11 | LSP1 | 0.10 (-0.03, 0.23) | 0.14 | 0.16 (-0.02, 0.33) | 0.08 | -0.08 (-0.27, 0.11) | 0.40 |
| rs703556 | genotyped | 12 | 138kb 5' of IGF1 | 0.67 (0.21, 1.12) | 0.004 | 0.79 (0.19, 1.40) | 0.01 | -0.36 (-1.03, 0.31) | 0.29 |
| rs1265507 | genotyped | 12 | 18kb 3' of LOC255480 | 0.07 (-0.05, 0.19) | 0.23 | 0.11 (-0.05, 0.27) | 0.16 | 0.04 (-0.13, 0.22) | 0.63 |
| rs7289126 | genotyped | 22 | TMEM184B | -0.03 (-0.15, 0.09) | 0.59 | 0.01 (-0.15, 0.17) | 0.89 | 0.07 (-0.11, 0.25) | 0.43 |
| rs17001868 | genotyped | 22 | SGSM3 | -0.01 (-0.18, 0.16) | 0.93 | -0.14 (-0.36, 0.09) | 0.23 | -0.24 (-0.48, 0.01) | 0.06 |
|  |  |  |  |  |  |  |  |  |  |
| ^a^square-root transformed  ^b^adjusted for study, reference age, case status, former use of MHT, BMI, number of pregnancies and principal components | | | | | | | | | |
