# Supplementary material for: A comprehensive evaluation of interaction between genetic variants and use of menopausal hormone therapy on mammographic density
Source: Breast Cancer Res. 2015 Aug 16;17(1):110. doi: 10.1186/s13058-015-0625-9 (PMC4537547; doi:10.1186/s13058-015-0625-9)
Supplement: Additional file 9: Figure S1. — University of California Santa Cruz (UCSC) Genome browser view (chr16:81958200–81963700) showing the intergenic position of single nucleotide polymorphisms (SNPs) rs7192724, rs17202296, and rs4888190, and correlated SNPs (r2>0.6 in 1000 Genomes CEU Pilot population). (DOC 231 kb) [file 13058_2015_625_MOESM9_ESM.doc]

**Supplementary Figure 1.** UCSC Genome browser view (chr16:81958200-81963700) showing the intergenic position of SNPs rs7192724, rs17202296, and rs4888190 (shown in green), and correlated SNPs (r^2^>0.6 in 1000 Genomes CEU Pilot population).
