# Supplementary material for: A comprehensive evaluation of interaction between genetic variants and use of menopausal hormone therapy on mammographic density
Source: Breast Cancer Res. 2015 Aug 16;17(1):110. doi: 10.1186/s13058-015-0625-9 (PMC4537547; doi:10.1186/s13058-015-0625-9)
Supplement: Additional file 10: Figure S2. — University of California Santa Cruz (UCSC) Genome browser view (chr6:22308200–22348900) showing the position of single nucleotide polymorphisms (SNPs) rs9356811, rs10946546, rs9358531, rs9393273, rs12525289, rs12199382 and rs12524161. (DOC 261 kb) [file 13058_2015_625_MOESM10_ESM.doc]

**Supplementary Figure 2.** UCSC Genome browser view (chr6:22308200-22348900) showing the position of SNPs rs9356811, rs10946546, rs9358531, rs9393273, rs12525289, rs12199382 and rs12524161 (shown in green).
